# Supplementary material for: Comparative multiomics analysis of cell physiological state after culture in a basket bioreactor
Source: Sci Rep. 2022 Nov 23;12:20161. doi: 10.1038/s41598-022-24687-4 (PMC9686226; doi:10.1038/s41598-022-24687-4)
Supplement: Supplementary file 1 — Supplementary Information 1. [file 41598_2022_24687_MOESM1_ESM.zip › raw data/Proteomic raw data/6.KEGGenrichment/EG.vs.CG/ALL/EG.vs.CG.KEGG_Enrich.scatterplot.pdf]

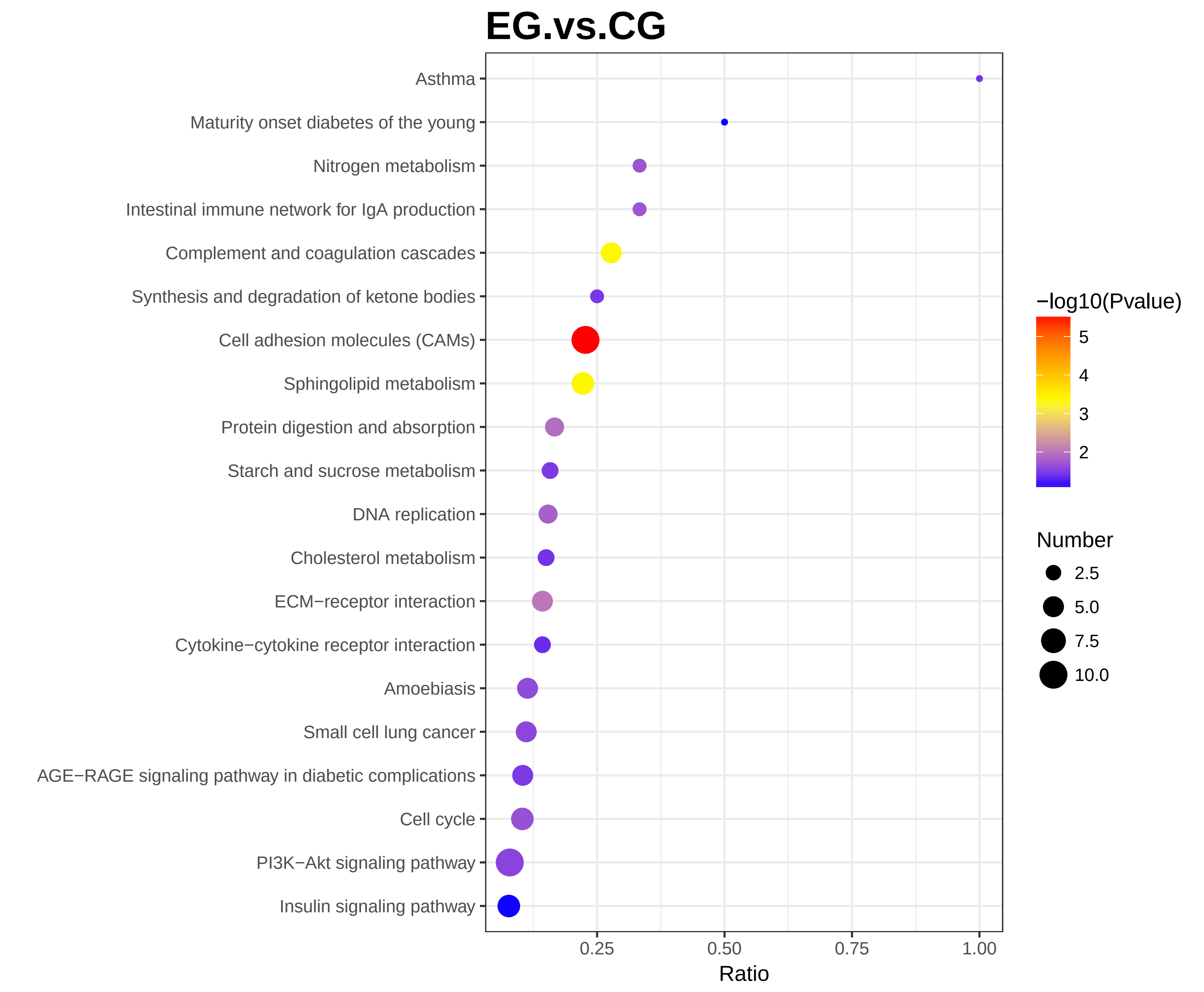
EG.vs.CG
Asthma
Maturity onset diabetes of the young
Nitrogen metabolism
Intestinal immune network for IgA production
Complement and coagulation cascades
Synthesis and degradation of ketone bodies
Cell adhesion molecules (CAMs)
Sphingolipid metabolism
Protein digestion and absorption
Starch and sucrose metabolism
DNA replication
Cholesterol metabolism
ECM−receptor interaction
Cytokine−cytokine receptor interaction
Amoebiasis
Small cell lung cancer
AGE−RAGE signaling pathway in diabetic complications
Cell cycle
PI3K−Akt signaling pathway
Insulin signaling pathway
0.25
0.50
0.75
1.00
Ratio
−log10(Pvalue)
5
4
3
2
Number
2.5
5.0
7.5
10.0
